# Self-Reported Maternal Parenting Stress From 9 m Is Longitudinally Associated With Child ADHD Symptoms at Age 12: Findings From a Population-Based Birth Cohort Study

**DOI:** 10.3389/fpsyt.2022.806669

**Published:** 2022-04-28

**Authors:** Kaori Endo, Daniel Stanyon, Syudo Yamasaki, Miharu Nakanishi, Junko Niimura, Sho Kanata, Shinya Fujikawa, Yuko Morimoto, Mariko Hosozawa, Kaori Baba, Nao Oikawa, Naomi Nakajima, Kazuhiro Suzuki, Mitsuhiro Miyashita, Shuntaro Ando, Mariko Hiraiwa-Hasegawa, Kiyoto Kasai, Atsushi Nishida

**Affiliations:** ^1^Research Center for Social Science and Medicine, Tokyo Metropolitan Institute of Medical Science, Tokyo, Japan; ^2^Department of Psychiatric Nursing, Tohoku University Graduate School of Medicine, Miyagi, Japan; ^3^Department of Psychiatry, Teikyo University School of Medicine, Tokyo, Japan; ^4^Department of Neuropsychiatry, Graduate School of Medicine, The University of Tokyo, Tokyo, Japan; ^5^Department of Psychology, Ube Frontier University, Yamaguchi, Japan; ^6^Institute for Global Health Policy Research, Bureau of International Health Cooperation, National Center for Global Health and Medicine, Tokyo, Japan; ^7^Graduate School of Nursing Science, St. Luke's International University, Tokyo, Japan; ^8^Department of Pediatrics, Juntendo University Faculty of Medicine, Tokyo, Japan; ^9^Department of Community Mental Health, Shinshu University School of Medicine, Matsumoto, Japan; ^10^Schizophrenia Research Project, Department of Psychiatry and Behavioral Science, Tokyo Metropolitan Institute of Medical Science, Tokyo, Japan; ^11^School of Advanced Science, SOKENDAI (Graduate University for Advanced Studies), Kanagawa, Japan; ^12^International Research Center for Neurointelligence (WPI-IRCN), The University of Tokyo Institutes for Advanced Study (UTIAS), Tokyo, Japan

**Keywords:** maternal parenting stress, ADHD, birth cohort, Maternal and Child Health handbook, adolescent

## Abstract

**Background:**

Attention-deficit/hyperactivity disorder (ADHD) develops in early childhood and carries lifelong impact, but early identification and intervention ensure optimal clinical outcomes. Prolonged or excessive parenting stress may be a response to infant behavioral differences antecedent to developmental disorders such as ADHD, and therefore represents a potentially valuable inclusion in routine early-life assessment. To investigate the feasibility of using routinely-collected self-reported maternal parenting stress as a risk marker for child ADHD, this study investigated the longitudinal association between maternal parenting stress from 1 to 36 months after childbirth and child ADHD in early adolescence.

**Methods:**

The sample comprised 2,638 children (1,253 girls) from the Tokyo Teen Cohort population-based birth cohort study. Mothers recorded parenting stress five times from 1 to 36 months following childbirth in the Maternal and Child Health Handbook, a tool used for routine early-life assessment in Japan. Nine years later, mothers evaluated their child's ADHD symptoms at 12 y using the hyperactivity/inattention subscale from the Strength and Difficulties Questionnaire.

**Results:**

Approximately 7.5% of parents reported that they had parenting stress at 36 m after childbirth. 6.2% of children were evaluated as above the cut-off for ADHD symptoms at 12 y. Parenting stress at 1 and 3–4 m was not associated with child ADHD symptoms at 12 y. However, child ADHD symptoms at 12 y was significantly associated with parenting stress at 9–10 m (unadjusted OR = 1.42, *p* =.047, 95% CI [1.00, 2/00]), 18 m (unadjusted OR = 1.57, *p* =.007, 95% CI [1.13, 2.19]) and 36 m (unadjusted OR = 1.67, *p* =.002, 95% CI [1.20, 2.31]). These associations remained after adjustment for child's sex, age in months and family income.

**Conclusions:**

We identified associations between parenting stress at 9–10, 18 and 36 m after childbirth and child ADHD symptoms at 12 years old. Self-reported parenting stress data may have utility as an early indicator for ADHD risk. Participation in early-life health checks, assessment of parenting stress, and tailoring support to family needs should be promoted for early identification and intervention for ADHD.

## Introduction

Attention-deficit/hyperactivity disorder (ADHD) is a psychological disorder impacting individuals across the lifespan, and may lead to poorer health outcomes such as depression, psychotic disorder, attempted suicide, and completed suicide (1–3). Approximately 30% of people with ADHD in childhood experience persisting symptoms in adulthood (1). While ADHD is a chronic condition, early identification and intervention have been shown to greatly improve outcomes. For example, interventions for preschool and school-aged children may facilitate social skills and reduce behavioral problems (4, 5). Additionally, parent training has demonstrated benefits for both child, via reduced internalizing and externalizing behaviors, and parent, via increased confidence and decreased parenting stress (6, 7).

Parenting a child is often stressful for parents in general, but parenting children with developmental difficulties may be especially challenging (8). A previous meta-analysis suggested that parents of children with ADHD symptoms experience significantly more parenting stress compared to parents of children without ADHD symptoms (9). Although early precursors of ADHD may be expressed as early as 3–18 months (10–14), ADHD is usually only considered for diagnosis once a child begins pre-school (15). As a result, parents caring for very young children with early ADHD symptoms may endure increased stress for prolonged periods before detection by support services. Prolonged parenting stress may lead to poorer parent mental health outcomes and harsh parenting strategies, both of which are reciprocally related to child ADHD symptom severity (16–18). Therefore, measuring parenting stress could have utility as both an early-life ADHD risk indicator and a signal to health professionals to provide relevant support for caregivers and their child before this cycle begins.

In the present study, we wished to investigate whether parenting stress could be clinically useful as a broad screening method for childhood ADHD risk. Using data from the Tokyo Teen Cohort (TTC), a prospective population-based birth cohort study in Japan (*N* = 3171), we investigated longitudinal associations between maternal parenting stress during the first 3 years following childbirth and child ADHD at age 12 within a community sample. To ensure clinical feasibility of potential screening, we used the Maternal and Child Health handbook (MCH), which is an already widely-adopted and routinely-used tool for infant health assessment in Japan. The MCH collects parenting stress data via a single self-report item and thus presents minimal time or response burden.

## Materials and Methods

### Participants

We used data from the TTC study (19), which is a population-based birth cohort study of child health and development using data from children and their caregivers. For the first wave of this cohort study, 3171 households with a child aged 10 were randomly sampled using the resident register from three municipalities (Setagaya, Chofu, and Mitaka) in Tokyo, Japan. For the second wave (age 12), 3,007 households participated (follow-up rate: 94.8%). At both waves, trained interviewers obtained written informed consent from the child's primary caregivers. As part of TTC's wider data collection procedure, participants were asked to complete a set of questionnaires. The study protocol of TTC was approved by the institutional review boards from the Tokyo Metropolitan Institute of Medical Science (Approval number [12–35]), SOKENDAI (Graduate University for Advanced Studies [2012002]), and the University of Tokyo [10057].

### Measurements

Participants were asked to fill in anonymous self-report questionnaires including questions about ADHD symptoms and sociodemographic characteristics (child's sex, age in months, and family income). Participants were also requested to report the responses recorded in their Maternal and Child Health handbook (MCH) on maternal parenting stress.

### Parenting Stress

The MCH handbook is a booklet distributed to newly pregnant women in Japan to facilitate routine assessment of child development and health by mothers as well as healthcare professionals (20). In 2018, 98% of all pregnant women reported receiving the MCH handbook within the first 20 weeks of pregnancy (21). The MCH handbook is used when mothers and their children attend health check-ups offered by the local health center or pediatric clinics, scheduled at regular intervals coinciding with developmental milestones (1, 3–4, 9–10, 18, and 36 months). In advance of each check-up, mothers answer the following question: “As a result of parenting, are there times when you feel distressed or find it difficult to cope?” Mothers can choose one of the following three responses: “yes,” “no,” or “difficult to say”. In the analysis, these responses were reclassified into dichotomous categories: “no” or “yes/difficult to say,” since we assumed that “difficult to say” may reflect that respondents were experiencing parenting stress but were reluctant to indicate so. The primary caregiver copied the parenting stress information from MCH handbook on the self-report questionnaire at the first wave of TTC (age 10).

### ADHD

Child ADHD symptoms were assessed using the hyperactivity/inattention subscale from the parent-report Strength and Difficulties Questionnaire (SDQ) (22, 23) at the second wave of the TTC study (age 12). The subscale consists of two items for inattention, two items for hyperactivity, and one item for impulsiveness (24). The three possible responses were “not true” [0], “somewhat true” (1), and “certainly true” (2). The responses from these five items were summed to produce a score from 0 to 10, with higher scores reflecting greater ADHD symptom burden. This subscale offers good predictive and discriminant validity across gender and age groups in adolescence (24). A cut-off value of 7 points or more was used for indicating high risk of ADHD, in line with previous studies (25–27).

### Sociodemographic Characteristics

Child age in months (calculated from birth date and survey response date), sex and socio-economic status (measured via family income) were adjusted for in analyses as potential confounders. Family income was categorized into 0–5, 5–10, and 10+ million yen per year, representing the lower, middle and upper thirds of household income distribution in Tokyo.

### Analysis

Bivariate binomial logistic regression analysis was used to examine the association between the presence of parenting stress at 1, 3–4, 9–10, 18, and 36 m, and child ADHD symptoms at age 12. Multiple binomial logistic regression analysis adjusting for sex, age in months, and annual household income followed for the above analysis. A full information maximum likelihood (FIML) estimation procedure was adopted to handle missing data (28) under the assumption of missing at random (MAR). All analyses were performed in Mplus 8.4.

## Results

Of the 3,007 households that participated the second wave (age 12) survey of the TTC study, 353 were excluded for the following reasons: did not own an MCH handbook, could not confirm whether they possessed an MCH handbook, had not responded to any items in the MCH handbook regarding parenting stress, had not reported child ADHD symptoms at age 12. Thus, the final analysis included 2,654 households (88.2%). Among the 2,654 cases, about half (52.5%) of children were boys. The mean age in months at the second survey wave was 145.9 months. 64.5% of participating households had annual household incomes below 10 million yen. Among 3,007 participants, more than 70% recorded parenting stress at all timepoints (73.4% at 1, 86.4% at 3–4, 85.3% at 9–10, 85.5% at 18, and 85.4% at 36 months).

As shown in Table 1, the prevalence of mothers reporting parenting stress was highest at 1 m (41.4%) and lowest at 9–10 m (26.5%). 6.1% of children had SDQ attention/hyperactivity scores indicating high risk of ADHD symptoms at age 12.

**Table 1:** Prevalence of maternal parenting stress during the first 3 years following childbirth, child ADHD at age 12, and demographic characteristics at age 12.

		***n***	**(%)**	***N***
Presence of maternal parenting stress^a^				
At 1 month of child's age		913	41.4%	2,206
At 3–4 months of child's age		822	31.7%	2,597
At 9–10 months of child's age		680	26.5%	2,565
At 18 months of child's age		779	30.3%	2,570
At 36 months of child's age		800	31.1%	2,569
Child's ADHD symptoms^b^ at age 12		163	6.1%	2,654
Demographic characteristics				
Boys		1,393	52.5%	2,654
Child age in months at 12 y [mean/SD]		145.90	3.62	2,654
Family income at 12 y, million yen per year				
	0–5	392	16.3%	2,399
	5–10	1,155	48.1%	
	10+	852	35.5%	

*N, Number of valid responses*.

a *Maternal parenting stress assessed using single self-report item from the Maternal and Child Health handbook (MCH)*.

b *Child ADHD assessed using the hyperactivity/inattention subscale from caregiver-report Strength and Difficulties Questionnaire (SDQ)*.

Table 2 shows the result of binomial logistic regression analysis of ADHD symptoms at age 12 from maternal parenting stress during the first 3 years after childbirth. At 1 m and 3–4 m, associations between parenting stress and ADHD symptoms at age 12 were not significant. However, ADHD symptoms at age 12 was significantly associated by parenting stress at 9–10 m (OR = 1.42, *p* = 0.047, 95% CI [1.00, 2.00]), 18 m (OR = 1.57, *p* = 0.007, 95% CI [1.13, 2.19]) and 36 m (OR = 1.67, *p* = 0.002, 95% CI [1.20, 2.31]) with increasing strength at each time point. After adjustment for child's sex, age in months, and annual household income, ADHD symptoms at age 12 remained significantly associated by parenting stress at 9–10 m (OR = 1.43, *p* = 0.048, 95% CI [1.00, 2.05]), 18 m (OR = 1.57, *p* = 0.010, 95% CI [1.11, 2.21]) and 36 m (OR = 1.64, *p* = 0.005, 95% CI [1.16, 2.30]) while associations between parenting stress at 1 and 3–4 m and ADHD symptoms at age 12 remained insignificant.

**Table 2:** Logistic regressions ofchild ADHD at age 12^a^ from maternal parenting stress during first 3 years following childbirth.

	**Unadjusted**					**Adjusted^c^**				
	**OR**	**95%CI**			***p***	**OR**	**95%CI**			***p***
Presence of maternal parenting stress^b^										
At 1 month of child's age	1.13	0.78	-	1.61	0.524	1.11	0.76	-	1.62	0.601
At 3–4 months of child's age	1.02	0.73	-	1.44	0.900	1.04	0.73	-	1.48	0.833
At 9–10 months of child's age	**1.42**	**1.00**	**-**	**2.00**	**0.047**	**1.43**	**1.00**	**-**	**2.05**	**0.048**
At 18 months of child's age	**1.57**	**1.13**	**-**	**2.19**	**0.007**	**1.57**	**1.11**	**-**	**2.21**	**0.010**
At 36 months of child's age	**1.67**	**1.20**	**-**	**2.31**	**0.002**	**1.64**	**1.16**	**-**	**2.30**	**0.005**

*OR, odds ratio; CI, confidence interval. Bold text indicates p < 0.05*.

*^a^Child ADHD assessed using the hyperactivity/inattention subscale from caregiver-report Strength and Difficulties Questionnaire (SDQ)*.

b *Maternal parenting stress assessed using single self-report item from the Maternal and Child Health handbook (MCH)*.

c *Adjusted for sex, age in months, and family income at age 12*.

## Discussion

To our knowledge, this is the first study to investigate the longitudinal association between maternal parenting stress at multiple intervals from 1 to 36 months post-childbirth and child ADHD symptoms at 12 years old in a population-based birth cohort sample. Our analyses found that parenting stress at 9–10, 18 and 36 months was associated (with subsequent increasing strength) with child ADHD symptoms at 12 years old, though parenting stress at earlier time points showed no such association. This finding suggests that self-reported parenting stress from 9 months may be a useful measure for early identification of children at risk of ADHD.

Our finding that parenting stress at 9–10 months associated ADHD symptoms in adolescence, with increasing strength at 18 and 36 months, raises the question of what differences emerge from 9 to 36 months in children who later develop ADHD that underlie this increase in parenting stress. Sleep disturbances between 0 and 5 years are associated with ADHD in adolescence (29, 30), and are associated with poorer parent mental health (31). Recent studies have also found heightened emotional reactivity at 6 and 9 months to be associated with ADHD, suggesting temperamental differences may emerge around this period (11, 12). The increasing strength of the relationship at 18 and 36 months may reflect that as motor development progresses and the child becomes more mobile, behavior manifesting core ADHD symptoms (e.g., hyperactivity and attentional difficulties) begins to emerge and impact more strongly on parents' wellbeing. Further research with young children with ADHD is necessary to determine how and what developmental differences emerge at this early age.

Parenting stress is a contributor to the increased likelihood of parents of children with ADHD using harsh or negative parenting strategies (16, 32), which may in turn exacerbate externalizing behaviors and other deleterious outcomes associated with ADHD (16, 33), highlighting the pressing need for early intervention. Several meta-analyses have demonstrated that behavioral parent training (PT) may reduce parenting stress, lead to positive parenting, and improve long-term outcomes for both the child and the parent (34–37). PT may confer reduced benefits when the parent themselves has ADHD (38, 39), though a more recent study found limited impact of parental ADHD symptoms on treatment outcomes (40). In either case, since ADHD is a highly heritable condition (41), health practitioners should ensure parental treatment needs (if any) are addressed alongside those of the child to ensure optimal outcomes for both.

This study shows the utility of the MCH handbook (and by extension, single-item self-report measures of parenting stress) for early identification of ADHD risk. This builds on a recent study examining the possibility of early identification of autism via developmental delay recorded in the MCH handbook (42) to encourage utilization of MCH data for early screening of mental health problems. We recommend that health professionals working with families aim to routinely capture parenting stress information, and specifically target parenting stress reduction when building family-specific support programmes.

Our study has a number of strengths. First, we used a large, representative population-based birth cohort, giving substantial ecological validity to our findings and allowing us to control for common sociodemographic confounders. Using the MCH handbook gave access to parenting stress data at frequent intervals over the first 36 months. In addition, since MCH handbook data was collected concurrently with infant health check-ups, our retrospective study design avoids the problem of recall bias. However, this study also had some limitations. First, we did not have parenting stress data beyond 36 months, as the MCH handbook does not record parenting stress after this time point. As such, we were unable to determine if this trend of increasing strength of parenting stress on adolescent ADHD symptoms persists as the child ages; this would be a potentially beneficial target for future research. Second, we used parent-reported child ADHD symptoms, rather than researcher-conducted interviews, teacher ratings, or clinical diagnosis. However, the SDQ attention/hyperactivity subscale has robust psychometrics for detecting ADHD (24, 43, 44), which may substantially offset this limitation. Lastly, the presence of parenting stress was captured by a binary-response question used in the MCH handbook, and since there is high social pressure on parenting success there may be a social desirability bias in response patterns. To get a more detailed picture of how parenting stress relates to child ADHD symptoms, future studies may consider using scales such as the Parenting Stress Index (45). This is particularly prescient as many developmental differences in temperament and behavior associated with ADHD may not be disorder-specific; for example, many are also associated with autism (13, 46). Future studies seeking to understand mechanisms or specificity of associations with early parenting stress may therefore benefit from including measures of autistic traits or other developmental differences in their analyses, either as control variables or additional outcomes of interest.

In conclusion, our study found that maternal parenting stress from 9 months post-childbirth was associated with child ADHD symptoms at age 12, with increasing strength at 18 months and 36 months. Maternal parenting stress from 9 months may be an indicator of later child ADHD. This time period may be extremely valuable for early intervention to ensure optimal outcomes in families caring for a child with ADHD.

## Data Availability Statement

The original contributions presented in the study are included in the article/Supplementary Material, further inquiries can be directed to the corresponding author.

## Ethics Statement

The studies involving human participants were reviewed and approved by Tokyo Metropolitan Institute of Medical Science. Written informed consent to participate in this study was provided by the participants' legal guardian/next of kin.

## Author Contributions

AN, KE, and DS conceptualized and designed the study, drafted the initial manuscript, and reviewed and revised the manuscript, being supervised by KK, MH-H, SA, and MN. JN, SK, SF, YM, MH, KB, NO, NN, KS, and MM critically reviewed the manuscript for important intellectual content and contributed to the discussion. SY supervised the statistical analysis. All authors contributed to and have approved the final manuscript.

## Funding

This work was supported by Grant-in-Aid for Transformative Research Areas (21A101) and Grant-in-Aid for Scientific Research on Innovative Areas (JP23118002 and JP16H01689; Adolescent Mind & Self-Regulation) from the Ministry of Education, Culture, Sports, Science and Technology of Japan. This study was also supported in part by JSPS KAKENHI (Grant Nos. JP16H06395, JP16H06398, JP16H06399, JP16K15566, JP16K21720, JP17H05931, JP19K17055, JP19H04877, JP20H01777, JP20H03951, JP21H05171, JP21H05173, and JP21H05174), JST-Mirai Program (Grant No. JPMJMI21J3); AMED (Grant No. JP17ek0109262); the UTokyo Center for Integrative Science of Human Behavior (CiSHuB); and the International Research Center for Neurointelligence (WPI-IRCN) at The University of Tokyo Institutes for Advanced Study (UTIAS). The funding source had no role in the preparation, review, or approval of the manuscript, and the decision to submit the manuscript for publication.

## Conflict of Interest

The authors declare that the research was conducted in the absence of any commercial or financial relationships that could be construed as a potential conflict of interest.

## Publisher's Note

All claims expressed in this article are solely those of the authors and do not necessarily represent those of their affiliated organizations, or those of the publisher, the editors and the reviewers. Any product that may be evaluated in this article, or claim that may be made by its manufacturer, is not guaranteed or endorsed by the publisher.
