# Supplementary material for: Self-Reported Maternal Parenting Stress From 9 m Is Longitudinally Associated With Child ADHD Symptoms at Age 12: Findings From a Population-Based Birth Cohort Study
Source: Front Psychiatry. 2022 Apr 28;13:806669. doi: 10.3389/fpsyt.2022.806669 (PMC9097942; doi:10.3389/fpsyt.2022.806669)
Supplement: Supplementary file 1 [file Table_1.docx]

**Supplementary table**

Logistic regressions of child ADHD symptoms at age 12 ^a.^ from maternal parenting stress during first three years following childbirth

|  |  |  | Unadjusted | | | | |  | Adjusted^c.^ | | | | |
| --- | --- | --- | --- | --- | --- | --- | --- | --- | --- | --- | --- | --- | --- |
|  |  |  | OR | 95%CI | | | *p* |  | OR | 95%CI | | | *p* |
| Presence of maternal parenting stress^b.^ | | |  |  |  |  |  |  |  |  |  |  |  |
|  | At 1 month |  |  |  |  |  |  |  |  |  |  |  |  |
|  |  | Yes | 1.03 | 0.58 | - | 1.83 | 0.912 |  | 0.96 | -0.65 | - | 0.55 | 0.882 |
|  |  | Difficult to say | 1.16 | 0.78 | - | 1.73 | 0.457 |  | 1.17 | -0.26 | - | 0.58 | 0.450 |
|  | At 3-4 months | |  |  |  |  |  |  |  |  |  |  |  |
|  |  | Yes | 1.12 | 0.63 | - | 2.00 | 0.697 |  | 1.08 | 0.59 | - | 1.98 | 0.792 |
|  |  | Difficult to say | 0.99 | 0.68 | - | 1.45 | 0.959 |  | 1.02 | 0.69 | - | 1.51 | 0.907 |
|  | At 9-10 months | |  |  |  |  |  |  |  |  |  |  |  |
|  |  | Yes | 1.35 | 0.73 | - | 2.51 | 0.340 |  | 1.27 | 0.66 | - | 2.44 | 0.468 |
|  |  | Difficult to say | 1.44 | 0.99 | - | 2.10 | 0.059 |  | **1.49** | **1.01** | **-** | **2.20** | **0.047** |
|  | At 18 months | |  |  |  |  |  |  |  |  |  |  |  |
|  |  | Yes | 1.52 | 0.89 | - | 2.60 | 0.128 |  | 1.53 | 0.88 | - | 2.68 | 0.134 |
|  |  | Difficult to say | **1.59** | **1.10** | **-** | **2.29** | **0.013** |  | **1.58** | **1.08** | **-** | **2.31** | **0.018** |
|  | At 36 months | |  |  |  |  |  |  |  |  |  |  |  |
|  |  | Yes | **2.45** | **1.54** | **-** | **3.91** | **<.001** |  | **2.33** | **1.44** | **-** | **3.79** | **0.001** |
|  |  | Difficult to say | 1.40 | 0.96 | - | 2.04 | 0.081 |  | 1.39 | 0.94 | - | 2.05 | 0.103 |

OR: odds ratio, CI: confidence interval. **Bold text** indicates *p* < 0.05.

^a^ Child ADHD assessed using the hyperactivity/inattention subscale from caregiver-report Strength and Difficulties Questionnaire (SDQ).

^b^ Maternal parenting stress assessed using single self-report item from the Maternal and Child Health handbook (MCH).

^c^ Adjusted for sex, age in months, and family income at age 12.
